# Oxidative Stress as a Primary Risk Factor for Brain Damage in Preterm Newborns

**DOI:** 10.3389/fped.2018.00369

**Published:** 2018-11-29

**Authors:** Isabella Panfoli, Giovanni Candiano, Mariya Malova, Laura De Angelis, Valentina Cardiello, Giuseppe Buonocore, Luca A. Ramenghi

**Affiliations:** ^1^Department of Pharmacy, University of Genoa, Genova, Italy; ^2^Laboratory of Molecular Nephrology, IRCCS Istituto Giannina Gaslini, Genova, Italy; ^3^Neonatal Intensive Care Unit, IRCCS Istituto Giannina Gaslini, Genova, Italy; ^4^Department of Molecular and Developmental Medicine, University of Siena, Siena, Italy

**Keywords:** adenosine, biomarker, oxidative stress, prematurity, white matter lesions

## Abstract

The risk of oxidative stress is high in preterm newborns. Room air exposure of an organism primed to develop in a hypoxic environment, lacking antioxidant defenses, and subjected to hyperoxia, hypoxia, and ischemia challenges the newborn with oxidative stress production. Free radicals can be generated by a multitude of other mechanisms, such as glutamate excitotoxicity, excess free iron, inflammation, and immune reactions. Free radical-induced damage caused by oxidative stress appears to be the major candidate for the pathogenesis of most of the complications of prematurity, brain being especially at risk, with short to long-term consequences. We review the role of free radical oxidative damage to the newborn brain and propose a mechanism of oxidative injury, taking into consideration the particular maturation-dependent vulnerability of the oligodendrocyte precursors. Prompted by our observation of an increase in plasma Adenosine concentrations significantly associated with brain white matter lesions in some premature infants, we discuss a possible bioenergetics hypothesis, correlated to the oxidative challenge of the premature infant. We aim at explaining both the oxidative stress generation and the mechanism promoting the myelination disturbances. Being white matter abnormalities among the most common lesions of prematurity, the use of Adenosine as a biomarker of brain damage appears promising in order to design neuroprotective strategies.

## Introduction

Oxidative stress is the consequence of an imbalance in the ratio among pro-oxidants and anti-oxidants in the cell (1). Free radicals, i.e., molecules bearing unpaired electrons, non-radical Reactive Oxygen Species (ROS) and Reactive Nitrogen Species (RNS) are collectively called oxidants, as they can easily lead to radical chain reactions (Table 1). ROS/RNS are generated from metabolic redox reactions (2) mostly by the respiratory chain (3), but also by microsomal cytochrome P450 system and by the immune response (4). Antioxidants, either endogenously produced or exogenously assumed, include enzymes, vitamins, minerals, and other substances (summarized in Table 1), which act neutralizing the excess of free radicals and protecting the cells against the harmful effects of oxidants (1).

**Table 1:** Main oxidants and anti-oxidants.

**Oxidants**	**Anti-oxidants**
**Free radicals** Hydroxyl radical, superoxide peroxyl, lipid peroxyl	**Enzymes** Superoxide dismutase, catalase, glutathione peroxidase, glutathione reductase
**Reactive Oxygen Species (ROS)** Peroxide, singlet oxygen, hypochlorous acid and lipid peroxides	**Vitamins** A, C, E
**Reactive Nitrogen Species (RNS)** Hydrogen nitric oxide, nitrogen dioxide, nitrous acid, peroxynitrite, dinitrogen trioxide	**Minerals** Se, Mn, Cu and Zn
	**Other substances** glutathione, melatonin, thiols, coenzyme Q, acetylcysteine, carotenoids and flavonoids

*Table summarizes the most common oxidants and the main endogenous and exogenous antioxidants*.

When the production of ROS exceeds the antioxidant defenses, or antioxidant levels are low, as is the case in the preterm newborn, oxidative stress usually occurs to the detriment of all of the cellular macromolecules (5). In adults, oxidative stress is recognized as a major contributing factor to the pathogenesis of a number of cardiovascular and neurological diseases, malignancies, diabetes, aging, inflammation and others (2, 6), Despite these deleterious effects, low or moderate concentrations of free radicals are necessary for many fundamental cellular functions, including host defenses (7).

## Oxidative stress as pathogenic factor in the preterm infant

The oxidant/antioxidant status balance is a process that begins before birth (8), and premature infants are particularly susceptible to oxidative stress (9, 10). Most of the complications of prematurity, such as bronchopulmonary dysplasia (BPD), retinopathy of prematurity (ROP), necrotizing enterocolitis (NEC), intraventricular hemorrhage (IVH), periventricular leukomalacia (PVL), and punctate white matter lesions (PWML), appear related to oxidative stress (11, 12), mostly occurring due to a mismatch among the free radical production and the anti-oxidative capacity of the premature neonate (10). Accordingly, Saugstad hypothesized that all of these complications may belong to one entity, “the oxygen radical disease of neonatology” (13). This topic was recently reviewed by Buonocore et al. (14).

Birth exerts the challenge of a hyperoxic insult due to the sudden exposure to a normoxic environment (100 mmHg oxygen tension, PO_2_) of an organism primed to develop in a hypoxic (20–25 mmHg, PO_2_) environment as the womb is. For this reason, current indications on neonatal resuscitation highlight the importance of starting respiratory support using the lowest oxygen concentration to reduce the postnatal oxidative stress (15). A randomized trial performed on neonates of 24–34 weeks gestational age who received resuscitation demonstrated that the use of room air, instead of 100% O_2_ as the initial resuscitation gas resulted lower oxidative stress, decreasing respiratory morbidities (16).

Together with hyperoxia, other main risk factors for oxidative stress exposure in preterm infants are hypoxia, ischemia, infections, and immune response activation, mitochondrial dysfunction, Fenton reaction due to both free iron and endothelial cell damage (17). Hypoxia has been demonstrated to be a risk factor for oxidative stress in preterm newborns. In a study conducted on 34 hypoxic and 15 healthy preterm newborns, plasma concentration of hypoxanthine, total hydroperoxide (TH), and AOPP were assessed both in umbilical cord blood immediately after birth and in peripheral blood on postnatal day 7 (18). Levels of these markers were significantly higher in hypoxic newborn at birth and at day 7 than in the healthy controls. Interestingly, a significant increase in TH and AOPP levels in non-hypoxic preterm newborns at day 7 was also observed, indicating that oxidative stress also occurs in non-hypoxic babies (18).

Moreover, antioxidant defense mechanisms are incompletely developed or deficient in preterm newborns (19). Preterm infants show reduced antioxidant defense mechanisms, including decreased levels of vitamin E, β-carotene, melatonin, ceruloplasmin, transferrin, and erythrocyte superoxide dismutase (SOD) (10). In a study on 100 preterm and 100 full-term neonates, plasma levels of vitamin A, vitamin E, and catalase were found significantly lower while plasma level of MDA, a marker of lipid peroxidation, was significantly higher in the preterm than in the full-term newborns, especially in those ones who developed NEC or BPD (20). A prospective study evaluated the concentration of vitamin D, glutathione peroxidase, SOD, MDA, and AOPP on 31 term neonates with hypoxic-ischemic encephalopathy (HIE) in comparison to 30 healthy term neonates (21). It was found that Vitamin D level, GP, and SOD were statistically lower on the first day of life in the study group compared to controls, while MDA levels were significantly higher in the study group (21). Although to date it has been difficult to design effective antioxidant therapies (19), the possibility can be envisaged to use particular kinds of antioxidants, such as melatonin, and effective free radical scavenger (22–24) and to design prophylactic antioxidant therapies also before birth.

## Oxidative stress-related brain injury

Advances in neonatal care allow preterm neonates to survive (25), but especially the very-low-birth-weight infants (VLBW) are at high risk to develop brain gray (GM) and white matter (WM) maturational disturbances, which may lead to neurodevelopmental disabilities (26, 27).

A study conducted on 119 consecutive premature infants admitted to neonatal intensive care units demonstrated a significant reduction in both cerebral cortical and deep nuclear GM volume and a subsequent increase in cerebrospinal fluid assessed with brain magnetic resonance at term equivalent age (TEA), in preterm infants compared with term infants (28). Along with gestational age at birth, the major predictor of altered cerebral volumes was the presence of cerebral WM injury, that most significantly correlated to neurodevelopmental outcome (28).

Cerebral WM injury is a full-spectrum of lesions named periventricular leukomalacia (PVL), that occurs in two overlapping forms: cystic PVL, in which the periventricular focal necrosis is macroscopic and evolves to multiple cysts; and non-cystic PVL, in which the focal necrosis are microscopic and evolve principally to glial scars (29). Evidence of PVL is found in 25 to 75% of VLBW infants with neuropathological examination (10). The incidence of cystic PVL declined significantly starting from late nineties of last century, now occurring in a minority of infants with abnormal neurodevelopmental outcome (30). Contemporary cohorts of preterm survivors commonly display milder forms of injury, primarily diffuse white matter injury (DWMI) and punctate white matter lesions (PWML), that even though do not involve pronounced neuronal loss may be also associated with a clear WM damage and neurodevelopmental disabilities (29, 31, 32). DWMI and PWML are currently the most common causes of brain injury in preterm infants (33, 34). Signs of DMWI occurs in about 50% of very low birth weight infants (35), while more than 10% of premature infants <32 weeks develop lesions visible at MRI performed at term corrected age. Oxidative stress is among the main causes of PWML (36–41). In fact, the optimal concentration of oxygen for resuscitation of very preterm infants is currently strictly monitored (15). Risk factors for the development of PWML and for oxidative stress production are similar, including hyperoxia, hypoxia, ischemia-reperfusion, hemorrhage, and maternal/fetal inflammation (33). Inflammatory microglial response in cerebral white matter can generate free radicals (42). A number of epidemiological studies have shown an association between infections and cerebral palsy (43) and intrauterine T cell activation and risk of cerebral lesions (44). VLBW infants with neonatal sepsis were shown to have increased rates of cerebral palsy and WM lesions, by a large cohort study (45). Another mismatch among demand and supply in the premature babies would regard insulin-like growth factor 1 (IGF-1), a mitogenic hormone involved in growth and metabolism. Increased chemical energy demand but low IGF-1 concentrations characterize preterm birth, which appears associated with complications such as especially ROP (46, 47).

## Oligodendroglial precursor injury as the main cause of brain damage

It has been reported that brain injury mostly affects WM, being oligodendroglial death the most important cause of PVL and PWML (35, 48, 49). Studies on both human brain and animal models assessed that the developing oligodendrocyte (OL) is the principal cellular target (49, 50). All of the cited risk factors can cause toxicity to the oligodendroglial precursors. For example, proinflammatory cytokines produced in response to hypoxia and infection can become toxic to the oligodendroglial precursor cells (pre-OL) (43). Glutamate excitotoxicity and free radical injury have recently been implicated in pre-OL death (39). Free iron, in turn causing oxidative stress, contributes to the onset of the OL dysmaturation (18).

In addition, oxidative stress reduces the expression of differentiation-promoting genes, such as Olig1, Olig2, and Sox10 in pre-OL, and increases the expression of differentiation-inhibiting genes (ID2 and ID4), resulting in the interruption of OL maturation (51).

Although there is evidence of an imbalance between antioxidant and oxidants, the ultimate cause of oxidative stress and molecular bases for the maturation-dependent vulnerability of the pre-OL to injury in a window of time ranging from 30 to 34 weeks of gestational age is yet unknown. We have recently proposed a bioenergetics hypothesis, correlating the oxidative stress generation to the significant increase of plasma Adenosine (Ado) concentration observed in some VLBW infants (52). Ado production may be triggered by the oxygen challenge and the untimely sensory stimulation consequent to premature birth. In particular, the pain sensory pathways would be primarily triggered by the invasive procedures routinely performed in intensive care units. A prospective randomized controlled trial evaluated the reduction of procedural pain 150 preterm newborns (gestational age 27–32 weeks) both pharmacological and non-pharmacological treatments to reduce the procedural pain in preterm newborn (41). Moreover, our recent unpublished data demonstrate that Ado concentration at day 15 was significantly associated with brain WM lesions evidenced using MRI performed at TEA. The underlying mechanism leading to myelination disturbances of the premyelinating preOLs, would be the consequence of the signal conveyed by Ado, which is a potent promoter of the preOLs differentiation (48, 53, 54).

Free radical production consequent to hypoxia and ischemia/reperfusion, together with the low caloric intake after birth in the premature babies may cause a slowing down of the oxidative phosphorylation, diminishing high-energy compounds (18, 55).

## Future directions

Many authors accept the hypothesis that free radicals persists to the damage of the premature brain. Consequently, to prevent long-term sequelae of oxidative stress, it is necessary to early diagnosis the presence of an oxidative stress damage by a validate panel of biomarkers, which could also represent the first step in delineating potential therapeutic interventions. To date, different biomarkers have been proposed to measure oxidative stress in the newborn. Plasma prostanoids were validated as biomarkers of oxidative stress injury to neurons (56). Visfatin, an adipocytokine involved in oxidative stress was also proposed (57) as a new marker of oxidative stress in preterm newborns. Ado blood concentration at day 15 after birth (52) may represent a biomarker to foresee premature brain injury, but further studies are needed to assess its diagnostic value in preterm infants (58).

Recently, novel treatment strategies have been proposed to counteract damages induced by oxidative stress in preterm infants (see Table 2), including the Ado antagonist caffeine (68). Considering the cited low postnatal IGF-1 concentrations in preterm infants, associated to ROP and other complications, a supplementation with recombinant human IGF-1 and its binding protein rhIGFBP-3 has been suggested (47). The preterm hypoxic status has been addressed by administration of erythropoiesis-stimulating agents (ESAs) in particular erythropoietin (EPO), that was shown to display low plasma levels. ESAs reduced the need for blood cell transfusions and decreased rates of IVH, and NEC (69). However, although promising, early EPO administration was not recommended by a Cochrane Systematic Review, due to its limited benefits (69) and its beneficial effect appears to require further studies.

**Table 2:** Main antioxidant Treatments.

**Treatment**	**Mechanism of action**	**References**
Caffeine	Free radical scavenger and adenosine receptor antagonist; Anti-inflammatory and anti-apoptotic.	Endesfelder et al. (59)
Erythropoietin	Anti-apoptotic, anti-oxidative and anti-inflammatory with angiogenic and neurogenic effects.	Rangarajan et al. (60), Maiese et al. (61)
Melatonin	Direct scavenger of oxygen free radicals, particularly the hydroxyl radical; Indirect antioxidant via stimulation of antioxidant enzymes.	Reiter et al. (62), Gitto E et al. (63), Miller et al. (64), Vladan et al. (24)
Allopurinol	Decrease free radical formation; Xanthine oxidase inhibitor; Directly scavenging free radicals.	Kaandorp et al. (65), Van Bel F et al. (66)
Quercetin	Increases survival against oxidative insults in neuronal culture.	Dajas et al. (67)

*Table reports the most common strategies contrasting oxidative stress employed to protect preterm brain from white matter injuries*.

In conclusion, despite gaps still present in our knowledge of the mechanism of oxidative stress production in the pathogenesis of brain damage in the premature newborn, this organ remains at major risk especially for the prolonged vulnerability of white matter at certain gestational ages during which preterm newborns undergo intensive care treatment. There is a need for new and accurate neonatal biomarkers of brain injury that can foresee those babies at higher risk of developing brain injury thus needing neonatal neuroprotection, by new therapeutic interventions centered on reversal of the processes that promote dysmaturation, one of the more important being oxidative stress.

## Author contributions

Both LR and IP devised main conceptual ideas and outlines. IP took the lead in writing the manuscript. All authors provided critical feedback and discussed the manuscript.

### Conflict of interest statement

The authors declare that the research was conducted in the absence of any commercial or financial relationships that could be construed as a potential conflict of interest.
